# On the definition of stigma

**DOI:** 10.1111/jep.13684

**Published:** 2022-04-23

**Authors:** Martin M. Andersen, Somogy Varga, Anna P. Folker

**Affiliations:** ^1^ National Institute of Public Health University of Southern Denmark København K Denmark; ^2^ School of Culture and Society Aarhus C Denmark

**Keywords:** metaphysics, philosophy of medicine, practical reasoning, public health

## Abstract

**Background:**

There are thousands of papers about stigma, for instance about stigma's impact on wellbeing, mental or physical health. But the definition of stigma has received only modest attention. In “Conceptualizing stigma” from 2001, Link and Phelan offer a thorough and detailed definition of stigma. They suggest that there are six necessary conditions for stigma, namely labelled differences, stereotypes, separation, status loss and discrimination, power, and emotional reaction. This definition is widely applied in the literature but is left mainly uncriticized.

**Method:**

We submit the Link and Phelan definition of stigma to a systematic conceptual analysis. We first interpret, analyze and reconsider each of the six components in Link and Phelan's definition of stigma, and on the basis of these analyses, we secondly suggest a revised definition of stigma.

**Result:**

The Link and Phelan definition is thorough and detailed, but includes redundant components. These are status loss and discrimination, and emotional reaction.

**Conclusion:**

We suggest that groups, not individuals, are the target of stigma, though it is individuals who may be the victims of it. We suggest a revised definition of stigma that is more simple, precise, and consistent with the empirical literature on stigma; there is stigma if and only if there is labelling, negative stereotyping, linguistic separation, and power asymmetry.

## 1 INTRODUCTION

In a landmark article from 2001 titled “Conceptualizing stigma”, Bruce G. Link and Jo C. Phelan note a “dramatic” increase in the number of papers that contain the word stigma in their titles or abstracts from 1980 to 1999,^1^ and the growth of stigma research has since continued.^2^ ^,^^p.25^ Researchers in sociology, psychology, and public health investigate the micro‐ and macro‐level causes of stigma, its impact on the welfare,^3^ mental health, and physical health of individuals,^4^, ^5^ its role in maintaining social hierarchies, and the nature of measures that carry the potential to assist destigmatization.^6^

Given the amount of research being generated to explore stigma, it is somewhat surprising that the *definition* of stigma has received only modest attention. In “Conceptualizing stigma”, Link and Phelan offer a thorough and detailed definition of stigma, and their paper has become the standard reference in much of the literature. As of August 4, 2021, it has been cited 9,674 times according to Google Scholar and 4,012 times according to Scopus. We searched within the 4,012 Scopus citations of the paper, and applied the following search string: “stigma AND definition AND (criticism OR critical)”, and we got 199 results. A title‐ and abstract screening left us with only a few papers that—as an indirect aim—criticized the details of the Link and Phelan definition. Moreover, we also applied the search string “stigma AND definition” in Philpapers on August 4 and got six results, the title and abstract screening of which, however, left no papers critical toward the Link and Phelan definition. Several scholars have noted that the concept of stigma is defined inconsistently throughout the literature, and that it is measured with different instruments,^7^, ^8^, ^9^, ^10^ but a systematic conceptual analysis is missing. While the Link and Phelan definition is both thorough and novel, we believe it can be significantly improved. Accordingly, the aim of this paper is to submit the Link and Phelan definition to a critical conceptual analysis and contribute to the development of a definition that is more simple, precise, and consistent with the empirical literature on stigma.

## 2 THE LINK AND PHELAN DEFINITION OF STIGMA

Interestingly, Link and Phelan's motivation for suggesting their definition is “(…) the observation that stigma is defined in different ways by different investigators.”^1^ ^,^^p.364^ As they note, since the publication of Goffman's *Notes on the Management of Spoiled Identity* back in 1963, the literature on stigma has grown significantly. Link and Phelan rightly point out that researchers tend to deploy different definitions of stigma and that the situation calls for a comprehensive and thorough definition.

Goffman defines stigma as an “attribute that is deeply discrediting” and as something that reduces its bearer “from a whole and usual person to a tainted, discounted one.”^11^ ^,^^p.3^ The point of Link and Phelan is not that Goffman is wrong (on that), but rather that his definition only insufficiently captures the nature of stigma. Instead, Link and Phelan offer the following conceptualization:

(…)stigma exists when the following interrelated components converge. In the first component, people distinguish and label human differences. In the second, dominant cultural beliefs link labeled persons to undesirable characteristics—to negative stereotypes. In the third, labeled persons are placed in distinct categories so as to accomplish some degree of separation of “us” from “them.” In the fourth, labeled persons experience status loss and discrimination that lead to unequal outcomes. Finally, stigmatization is entirely contingent on access to social, economic, and political power that allows the identification of differentness, the construction of stereotypes, the separation of labeled persons into distinct categories, and the full execution of disapproval, rejection, exclusion, and discrimination.^1^ ^,^^p.367^

Notably, it takes five components to converge for there to be stigma. For them to “converge”, we take it that each component is a necessary condition for stigma. It follows that there is no stigma if not all of the five components are satisfied:

To the extent that we can answer yes to these questions, we can expect stigma to result. To the extent that we answer no, some of the cognitive components of stigma might be in place, but what we generally mean by stigma would not exist.^1^ ^,^^p.376^

Interestingly, in “Measuring Mental Illness Stigma” from 2004, Link, Yang, Phelan, and Collins expand the 2001 definition by adding a component of (individual negative) *emotional reaction* (on the stigma).^12^ ^,^^p.513^ We therefore consider Link and Phelan's (and partly Yang and Collins') definition of stigma to consist of six necessary conditions. For there to be stigma there must be labeled differences, stereotypes, separation, status loss and discrimination, power, and emotional reaction. It is worth adding thatLink and Phelan maintain that stigma exists as a matter of degree.^1^ ^,^^p.377^ The degree of separation can be more or less complete and the labeling can be more or less prominent, involving few or many, more or less negative, stereotypes, and these factors will have an influence on the extent of the resulting status loss and discrimination.

Now, we will first look into, interpret, and analyze each of these six components. On the basis of these analyses, we subsequently suggest a revised definition of stigma.

### 2.1 First and second component: Labeled differences and stereotypes

“Why is it that some human differences are singled out and deemed salient by human groups while others are ignored?”^1^ ^,^^p.368^ This fundamental sociological question frames the first component of Link and Phelan's definition of stigma. For there to be stigma there must be a social selection of human differences. There are countless differences between humans and their possessions, for instance in the number of fake Claude Monet paintings, eye colour and whether one likes to eat potatoes. Such differences seem harmless. Other differences, however, are somehow selected to matter, they are “(…) socially selected for salience (…).”^1^ ^,^^p.368^ Some differences are selected to be relevant, important, or significant. They are *labeled*.

Now such labeled differences tend to be linked to stereotypes. We all seem to do such linking as a matter of cognitive efficiency; we make fast judgments based on induction. When we have observed 50 white swans, we tend to expect the 51th swan to be white too. When we have seen four drunken homeless men, we tend to link homelessness to alcohol abuse. It is an open question when we are epistemically justified in such inductive reasoning. Sometimes we are, often we are not, but as we understand Link and Phelan, this is not important here. What is important is the effect on the homeless if we automatically associate them with alcohol abuse, if we automatically associate obesity with lack of will power, and so forth.

Thus, the basis of stigma lies in the linking of labeled differences to stereotypes. This also matches Goffman's definition. It is worth considering the fact that some labeled differences are linked to positive stereotypes, not negative. For instance, being tall is linked to power, and speaking French is linked to being sophisticated. But these, of course, are stereotypes; not all tall people have power, and not all French speakers are sophisticated. Importantly, such *positive* stereotypes cannot be components of stigma. Link and Phelan clearly state that the characteristics that form the stereotype must be *undesirable*.^1^ ^,^^p.369^ For clarity, we add that this should be understood as undesirable according to dominant societal norms and beliefs. Thus, it lies in the concept of stigma that it is an unpleasant thing, at least for those who are the victims of it. We agree with the first and second components of stigma as suggested by Link and Phelan.

### 2.2 The third component: Separation

The third component of stigma requires the label to connote a separation of “us” from “them”. Link and Phelan suggest that “(…) the linking of labels to undesirable attributes (…) become the rationale for believing that negatively labeled persons are fundamentally different from those who don't share the label (…).”^1^ ^,^^p.370^ While this component seems intuitive at first, a closer look suggests that it is actually superfluous, because it is already entailed in the labelled differences component. To label differences necessarily implies some separation of us and them, at least in a linguistic sense. We separate brown‐eyed from blue‐eyed, the tall from the short, the men from the women, the English from the French, and so forth. Separation is, in other words, a logical implication of labeled differences. But perhaps Link and Phelan are gesturing toward something narrower, like a particular type of separation. So, when more precisely does a labeled difference satisfy the component of separating us and them? Link and Phelan suggest that:

Evidence of efforts to separate us from them are sometimes directly available in the very nature of the labels conferred. Incumbents are thought to “be” the thing they are labeled (Estroff 1989). For example, some people speak of persons as being “epileptics” or “schizophrenics” rather than describing them as having epilepsy or schizophrenia.^1^ ^,^^p.370^

The distinction between *having X as an attribute* and *being X* is rhetorically very powerful. *Potato‐eaters* might have been stigmatized during history, but *those who enjoy potatoes* have not. Giving names to labeled differences linked to negative stereotypes seems evidence that the labeled difference is one that really matters. If someone really is your enemy, if you really want to distinguish yourself from someone, then you give them a name! *Those who smoke* are not the target of potential stigma, but *the smokers* are. Accordingly, we echo the separation component given the following linguistic interpretation: it is a necessary condition for a target to be stigmatized that it is commonly referred to by a name. Empirically, it can somewhat easily be verified if this component is satisfied in some social context.

### 2.3 Fourth component: Status loss and discrimination

The fourth component in Link and Phelan's stigma definition is *status loss and discrimination*. It is unclear why this is one and not two components, so let us look at status loss first and discrimination second.

#### 2.3.1 Status loss

Link and Phelan explain that: “An almost immediate consequence of successful negative labeling and stereotyping is a general downward placement of a person in a status hierarchy.”^1^ ^,^^p.371^ We humans create social hierarchies and evaluate one another from the perspective of such social hierarchies. We form social hierarchies on the basis of human differences that we consider relevant: level of education, income, age, looks, weight, height, and so forth. It is reasonable to think that the human differences we consider relevant when forming social hierarchies also are human differences that are socially salient and thus capable of constituting stigma. If one can be stigmatized because of one's weight, it is because weight is socially salient. And if weight is socially salient, it is also one among several other parameters that we use to form social hierarchies. Thus, echoing other papers of the same authors, the building blocks of status hierarchies seem to be the same as the building blocks of labeling and stereotyping.^5^, ^13^ As such it seems right that status loss is a consequence of successful negative labeling and stereotyping. However, a detail is worth considering.

Link and Phelan suggest that a consequence of labeling and stereotyping is “(…) a general downward placement of a person in a status hierarchy.” Elsewhere, however, they suggest that: “(…) people are stigmatized when the fact that they are labeled, set apart, and linked to undesirable characteristics leads them to experience status loss and discrimination (our underline).”^1^ ^,^^p.371^ So, is it the *experience of* or the *de facto* status loss that is the relevant currency? Well, a firefighter might *feel* stigmatized. But she isn't. At least not for *being a firefighter*, in any country we know of. The mere *feeling* of being stigmatized, whatever the reason, does not imply de facto stigmatization. And on the contrary, a homeless person with an alcohol abuse disorder might very well be stigmatized in many countries that we know of, but what if she does not feel the stigma? Would it then be stigma? Well, in 2004, Link, Yang, Phelan, and Collins expands the 2001 definition by adding a component of ‘emotional reaction’. Therefore, if the feeling of the stigma matters to stigma (we will consider this question when considering the component of emotional reaction), this seems to be accounted for by this added component. Accordingly, we interpret the criterion of status loss as de facto status loss, that is, in the eyes of society.

The notion of de facto status loss in the eyes of society raises difficult questions of causality and of quantification; what are the mechanisms behind status ranking? And how many individuals, and which particular groups, in the society must lower their ranking for it to be a status loss in the relevant sense? These interesting questions are beyond the scope of this paper. We should note, however, that even though someone is stigmatized, status loss is not necessarily entailed. This is so because many elements constitute our status. If, for instance, BMI > 35 is stigmatized, then persons gaining weight to BMI > 35 will lose status, *all else being equal*. But some of these individuals might at the same time change in ways that are status beneficial. If, for instance, some unemployed with BMI > 35 suddenly gets a new well‐paid job, then she will benefit status‐wise from the latter while losing status from the former. Therefore, we should say that status loss, *all else being equal*, is a consequence of labeling and stereotyping. Let us now consider if de facto status loss really is a necessary condition for stigma.

First, if the building blocks of labeling and negative stereotypes are the same as the building blocks of our social hierarchies, namely the same human differences, then one might think that status loss is a necessary consequence—a logical implication—of labeling and negative stereotypes. But, following the spirit of Occam's razor, if it is a necessary consequence, then there seems to be no reason for including it in the definition. Indeed, this consequence could be relevant for understanding the *problem* of stigma, but in the definition it would be redundant. By analogy, it is a necessary consequence of being obese that one does not have a body mass index (BMI) < 20, but there is no reason for including this implication in the definition of obesity.

Second, status loss could be a *contingent* consequence of labeling and negative stereotyping. If so, however, it would be wrong to add it to the definition. It would namely mean that there are both cases of stigma that have status loss as a consequence and cases of stigma that do not have status loss as a consequence. But if there are cases of stigma that do not have status loss as a consequence, then status loss is not necessary to stigma; it is not part what stigma is.

In sum, de facto status loss is, all else being equal, either a necessary consequence (a logical implication) or a mere contingent consequence of successful negative labeling and stereotyping. In either case it seems we should leave it out of the definition.

#### 2.3.2 Discrimination

The second part of the fourth component of stigma is *discrimination*. Unfortunately, discrimination itself is a complex concept and therefore it is not clear how we should understand this component. For, on the one hand, there is an obvious but also trivial way in which it seems right that the stigmatized are discriminated against. When a human difference is labelled, say, obesity, and linked to undesirable stereotypes, say, being lazy and having a lack of willpower, it seems to hold true that those who uphold this label and this stereotype look at *the obese* in light of this label and this stereotype. And as others, *those who are not obese*, will not be looked at in light of this label and this stereotype, there is discrimination; *the obese* are treated, at least looked upon, differently than the *not‐obese*. But if this is what we mean by discrimination, then it seems redundant as a component in the definition, because it is already implied by the labeling and stereotyping.

On the other hand, if Link and Phelan want more from the discrimination component than this, we need first realize that in one sense we are all discriminated against for one reason or another. We (the authors of this paper) are not nominated for any Oscar, but some filmmakers are, nondisabled persons are not welcome to compete against persons with disabilities at the Paralympic games, and so forth. For discrimination to be relevant, however, it should be discrimination that is morally (or juridically) wrong, or objectionable.^14^ For instance, it is not an interesting case of discrimination when people cannot receive unemployment benefits while being gainfully employed. But it is, when men and women at the same level of qualification receive unequal payment for the same work. This distinction holds true for the otherwise stigmatized too: when an obese person is simply unable to fit the standard airplane seat and is therefore offered a larger one, it is discrimination—the nonobese are not offered a larger seat—but hardly discrimination in any objectionable way. If unequal seat sizes are offered because of unequal sizes, not because of stereotypes, then it does not seem morally objectionable. On the other hand, if the best qualified for the job does not get the job because of obesity, then it is morally objectionable discrimination. However, it is a matter of deep disagreement what morality more precisely requires and therefore which discriminatory acts would count as morally wrong or objectionable. Link and Phelan suggest that a definition of stigma does not exist “(…) in some independent existential way. As such, its value rests in its utility.”^1^ ^,^^p.377^ This seems right. But it seems to count against its utility if our conceptualization of stigma hinges upon our agreement on when discrimination is morally objectionable and when it is not. This seems much too demanding.

So, should discrimination be left out of the definition of stigma? We suggest it should, though it is probably true that stigmatized people are very often discriminated against in morally (or juridically) objectionable ways. But there are thousands of empirical papers on the impact of stigma on wellbeing, mental health, and physical health. Only very few of them, if any, would be able to lift the burden of showing that the otherwise stigmatized are discriminated against in a morally (or juridically) objectionable way. If discrimination in any nontrivial sense is part of the stigma definition, then we cannot really know if it is stigma that all those studies are measuring. Thus, scientific utility seems to count against the component of discrimination, and as Harriet Deacon puts it in an indirect critique, separating discrimination from stigma “allows us to think more clearly about negative consequences of stigma (…).”^10^ ^,^^p.421^

### 2.4 Fifth component: The dependence on power

The fifth, and in the 2001 paper the last, component of stigma is power: “Stigma is entirely dependent on social, economic, and political power—it takes power to stigmatize”.^1^ ^,^^p.375^ The point of Link and Phelan is that whereas there might be labeling and stereotyping upward on the social hierarchy, it should only count as stigma when it goes downwards. They notice that mentally ill patients might label clinicians as e.g. “pill pushers” and link them to the stereotypes of being cold, paternalistic, and arrogant. But the clinicians will not, therefore, be a stigmatized group, because this group of patients simply do not possess the sufficient power to “(…) imbue their cognitions about staff with serious discriminatory consequences.”^1^ ^,^^p.376^

We very much agree with this component. It is the asymmetrical power relation between the stigmatizer and the stigmatized that makes the labeling and stereotyping matter. When it matters to be labeled and stereotyped, it is because it is done by someone who has the power to discriminate against you and in other ways treat you badly. Should individuals who smoke be alarmed about the label *smoker* and associated stereotypes from the nonsmokers? Fifty years ago the answer was in the negative, but today, in the western world at least, individuals who smoke have good reasons to be alarmed about the label and associated stereotypes, because the power balance has changed; individuals who smoke are overrepresented among lower socioeconomic groups, and the nonsmokers have the power to discriminate against the smokers and in other ways treat them badly.

Thus, we believe the power component is well‐put and indeed a necessary condition for stigma. Power, however, is a complicated matter, and there are several interesting questions about its nature and quantification that we cannot speak to here. For instance, about how we might have more power in some social contexts than in others. Whereas it seems right that the mentally ill as a group cannot stigmatize the psychiatrists because of power asymmetry, it does not follow that a psychiatrist cannot belong to a stigmatized group and thus be stigmatized by mentally ill persons and others. If obesity is stigmatized, then the obese psychiatrists will also be stigmatized. The relevant power balance seems here to be between the obese and the nonobese and even though some obese persons belong to higher socioeconomic groups, the nonobese have the power to discriminate against the obese. This raises the question of whether it is *groups*, *individuals*, or *both* that ultimately are the target of stigma. We will try and answer this below.

### 2.5 Sixth component: Emotional reaction

In “Measuring Mental Illness Stigma” from 2004, Link, Yang, Phelan, and Collins suggests adding a component of ‘emotional reaction’ to the 2001‐definition:

We believe that this underrepresentation needs to be corrected, because emotional responses are critical to understanding the behaviour of both stigmatizers and people who are recipients of stigmatizing reactions.^12^ ^,^^p.513^

The question is, should we accept something as stigma if the otherwise stigmatized individuals do not react upon it emotionally? Well, stigma leads to negative emotional reactions for many of its victims, but that does not show that emotional reaction is part of what stigma is. Rather, this sixth component somehow mixes up what stigma is and what often is a consequence of it. Thus, stigma's impact on wellbeing and health explains why we should care about stigma, why stigma is dangerous. But if we say that there must be (negative) emotional reaction for there to be stigma, why should we investigate empirically if stigma impacts wellbeing and health? It would already be part of the definition. Of course, one might say that we need the empirical investigations to merely confirm (or reject) that some labeling and negative stereotyping and so forth is in fact stigma. But if so, it would come with a heavy burden of proof to suggest that someone is stigmatized, because one would have to show that the person in question actually undergoes an emotional reaction because of being otherwise stigmatized. This also means that we would often be wrong in saying that some group, say *the smokers*, is stigmatized. We may be able to satisfy the components of labeling, negative stereotyping, separation, and asymmetrical power, but for the proposition to be true—that smokers are stigmatized—we would have to know that *every* individual belonging to the group actually has a (negative) emotional reaction. This does not seem fruitful.

There is an additional reason to reject the component of emotional reaction. A number of studies indicate that a significant proportion of people with mental disorders internalize stigma, meaning that they come to believe and apply to themselves stigmatizing suppositions and stereotypes linked to the mental disorder.^15^ While the process by which internalization occurs is complex, a brief reflection on a simplified case offers enough ground to challenge the proposition that emotional reaction is a necessary condition for stigma. Consider someone suffering from schizophrenia who internalizes the stigma by endorsing the relevant stereotype. While this person is obviously stigmatized, we expect that she would not react emotionally to the label and stereotype, because she thinks it is accurate. But then, our conclusion must be that emotional reaction is not a necessary component of stigma.

Accordingly, we suggest that emotional reaction is not a component in the definition of stigma. In fact, we mean to suggest that stigma is a social phenomenon in the sense that it is entire groups, not just some individuals in the group, that may or may not be stigmatized. But the damage is on the individual. Stigma is a social phenomenon; it is dangerous because it very often, but not by definition, leads to negative individual emotional reactions; often, but not by definition, it leads to discrimination, and so forth.

## 3 STIGMA DEFINITION REVISED

Stigma is a social phenomenon. But what is it more precisely? We have found no reason to question that it is *labeling, negative stereotyping*, linguistic *separation*, and that there must be *asymmetrical power*. But is this enough?

Perhaps it is worth considering what we want from the definition. As Link and Phelan suggest, the definition of stigma does not exist “(…) in some independent existential way. As such, its value rests in its utility.”^1^ ^,^^p.377^ We agree that the value of a definition of stigma (and indeed many other definitions) rests in its utility. But how are we to comprehend its utility? More generally, definitions are designed to advance understanding by providing a characterization of all entities or examples of a particular type. The definition of X is a statement that aims at expressing the essential nature of X in a way that includes all instances of X while excluding all instances that aren't really X. All definitions are supposed to give us the essence of the thing we want to define, but it is not always obvious what the essence is supposed to be. For instance, the chemist's definition of *gold* and the lexicographer's definition of *cool* both aim to achieve accuracy and to advance understanding, but while the chemist's definition should “cut nature at its joints”, the lexicographer's definition should truthfully reflect a linguistic practice. Or, put in Lockean terms, the lexicographer should aim at providing a “nominal definition”, which offers us a clearer view of our uses of the word “cool”, while the chemist should aim at offering a “real definition”, which aspires to give an account of what ‘gold’ is independent of our practices.^16^

Defining stigma seems to be a task somewhere in between these examples. Stigma is a thing that really matters to wellbeing, mental health, and physical health. Therefore its definition is more than a linguistic practice; stigma does have some essence which its definition is supposed to capture. Accordingly, here are five selective meta‐criteria of what a definition of stigma and many other social concepts, in our opinion, ought to satisfy. We do not mean to suggest that these criteria are all that matters to the plausibility of a definition, nor that they are equally important, only that they matter:

(1) the extent to which the definition matches its target, that is the thing it refers to;
(2) the extent to which the definition is applicable;
(3) the extent to which the definition matches the ordinary and/or scientific use of the concept;
(4) precision, meaning that all redundant and ambiguous parts should be eliminated; and
(5) consistency, meaning that the definition cannot have incompatible implications.

Now, let us revise the definition of stigma in light of these meta‐criteria. Acknowledging that stigma leads to status loss, should the definition contain this component? Well, either the status loss is a logical implication or a mere contingent consequence of successful negative labeling and stereotyping. If status loss is a logical implication, then it is redundant in much the same way as it would be redundant to include in a definition of obesity that the obese does not have a BMI > 20. If status loss, on the other hand, is a contingent consequence of labeling and negative stereotyping then it would be wrong to add it to the definition, because there would both be cases of stigma that have status loss as a consequence and cases of stigma that do not have status loss as a consequence. Therefore status loss fails on meta‐criterion 4 and 5.

Should there be a component of discrimination? For discrimination to be relevant, it should be discrimination that is morally (or juridically) wrong, or objectionable. But only very few studies measuring stigma would be able to lift the burden of showing that the otherwise stigmatized are discriminated against in a morally (or juridically) objectionable way. If discrimination in any nontrivial sense is a component of stigma, we cannot really know if it is stigma that all those studies are measuring. The applicability of the concept would seem unnecessarily low. The discrimination component fails on meta‐criteria 2 and 4.

Should there be a component of emotional reaction? If the individual would have to show some emotional reaction for her to be stigmatized, it would be a heavy burden of proof to suggest that someone is stigmatized; one would have to show that the person in question actually has some emotional reaction upon it. This also means that we would often be wrong in saying that some group, say, *the smokers*, is stigmatized. We may be able to satisfy the components of labeling, negative stereotyping, separation, and asymmetrical power, but for the proposition to be true—that smokers are stigmatized—we would in principle have to know that *every* individual belonging to the group actually has a (negative) emotional reaction. This does not seem fruitful. The component of emotional reaction seems to fail on meta‐criteria 2 and 3.

Thus, we suggest that there is stigma if and only if:

there is labelling,
negative stereotyping,
linguistic separation (the target is commonly referred to by a name), and
power asymmetry.

This definition implies that stigma is a social phenomenon. It is groups that are the target of stigma, but it is individuals who pay the price. It rests on the influential work of Link and Phelan, but is slimmed for redundances and inconsistencies. It isolates stigma from its consequences. It is more applicable in the sense that those who measure stigma in different ways can verify much more easily whether they measure the concept they aim at. And it more accurately captures our linguistic practices of referring to entire groups of individuals as being stigmatized.

## CONFLICTS OF INTEREST

The authors declare no conflicts of interest.

## Data Availability

Data sharing not applicable to this article as no datasets were generated or analysed during the current study. This manuscript has been accepted for the special issue on Health Philosophy
